# Plasma concentrations of SSRI/SNRI after bariatric surgery and the effects on depressive symptoms

**DOI:** 10.3389/fpsyt.2023.1132112

**Published:** 2023-04-25

**Authors:** Patrick Pasi, Dino Kröll, Alena Siegfried, Martin Sykora, Alessandro Wildisen, Cristiana Milone, Gabriella Milos, Laura Horka, Stefan Fischli, Christoph Henzen

**Affiliations:** ^1^Department of Consultation-Liaison Psychiatry and Psychosomatic Medicine, University Hospital Zürich, University of Zürich, Zürich, Switzerland; ^2^Division of Visceral Surgery, University Hospital Bern, University of Bern, Bern, Switzerland; ^3^Department of Surgery, Division of Visceral Surgery, Cantonal Hospital, Lucerne, Switzerland; ^4^Department of Internal Medicine, Division of Endocrinology, Cantonal Hospital, Lucerne, Switzerland

**Keywords:** selective serotonin reuptake inhibitor (SSRI), serotonin noradrenalin reuptake inhibitor (SNRI), laparoscopic Roux-en-Y gastric bypass (RYGB), sleeve gastrectomy (SG), Beck depression inventory (BDI), body mass index (BMI)

## Abstract

**Background:**

Depression and treatment with antidepressants SSRI/SNRI are common in people with morbid obesity who are candidates for bariatric surgery. There is few and inconsistent data about the postoperative plasma concentrations of SSRI/SNRI. The aims of our study were to provide comprehensive data about the postoperative bioavailability of SSRI/SNRI, and the clinical effects on depressive symptoms.

**Methods:**

Prospective multicenter study including 63 patients with morbid obesity and therapy with fixed doses of SSRI/SNRI: participants filled the Beck Depression Inventory (BDI) questionnaire, and plasma levels of SSRI/SNRI were measured by HPLC, preoperatively (T0), and 4 weeks (T1) and 6 months (T2) postoperatively.

**Results:**

The plasma concentrations of SSRI/SNRI dropped significantly in the bariatric surgery group from T0 to T2 by 24.7% (95% confidence interval [CI], −36.8 to −16.6, *p* = 0.0027): from T0 to T1 by 10.5% (95% 17 CI, −22.7 to −2.3; *p* = 0.016), and from T1 to T2 by 12.8% (95% CI, −29.3 to 3.5, *p* = 0.123), respectively.

There was no significant change in the BDI score during follow-up (−2.9, 95% CI, −7.4 to 1.0; *p* = 0.13).

The clinical outcome with respect to SSRI/SNRI plasma concentrations, weight change, and change of BDI score were similar in the subgroups undergoing gastric bypass surgery and sleeve gastrectomy, respectively. In the conservative group the plasma concentrations of SSRI/SNRI remained unchanged throughout the 6 months follow-up (−14.7, 95% CI, −32.6 to 1.7; *p* = 0.076).

**Conclusion:**

In patients undergoing bariatric surgery plasma concentrations of SSRI/SNRI decrease significantly by about 25% mainly during the first 4 weeks postoperatively with wide individual variation, but without correlation to the severity of depression or weight loss.

## Introduction

Depression is common in people with morbid obesity undergoing bariatric surgery. In a large meta-analysis depression was identified as the most common preoperative mental health condition, affecting almost 1 in 5 patients. However, regarding the association between preoperative depression and postoperative weight loss there was conflicting evidence (1). Longitudinal assessments revealed a positive effect of bariatric surgery on quality of life, mood (2, 3) and depression, with significant relation to change in body mass (4). However, depressive symptoms deteriorated again 1 year after bariatric surgery, and there are reports describing subgroups of patients with worsening of comorbid psychiatric disorders, increased risk of suicide, and lower long-term weight loss compared to control groups undergoing conservative treatments (5, 6).

Because treatments with psychotropic drugs are prevalent in people with morbid obesity before and after bariatric surgery, and SSRI/SNRI are the most commonly used antidepressants, knowing changes in pharmacokinetics and their impact on depressive symptoms is most important. However, little is known about the impact of bariatric surgery on psychotropic drug concentrations, and thereby potentially influencing efficacy and safety, including potential psychiatric consequences (e.g., relapse or deterioration of symptoms).

To the best of our knowledge, only two cross-sectional studies examined the pharmacokinetics of SSRI/SNRI, showing a significant decrease in the area under the curve (AUC) compared to a matched control group (7, 8). Two case series demonstrated similar results, however, restricted to a small number of patients, with a short postoperative follow-up, and without a control group. For example, Marzinke et al. found a 33% decrease of escitalopram concentrations with high interindividual variations in only four patients compared to postoperative values 2 weeks after bariatric surgery (9).

Hamad et al. (10) investigated the bioavailability of an SSRI test dose in 12 patients: serum concentrations were significantly reduced in 8 patients in the first month after gastric bypass, and normalized in 6 patients after 6 months, and in 4 patients after 12 months. However, 4 patients showed even higher SSRI levels than before surgery, with improvement in depression, recorded by the Structured Interview Guide for the Hamilton Depression Rating Scale-Atypical Depression Symptom Version (SIGH-ADS).

Patients lose weight significantly after bariatric surgery, leading to marked improvement of the metabolic syndrome (11), and some patients have a reduction or discontinuation of their antidepressant medications, and depressive symptoms tend to increase or often change after bariatric surgery (12).

Recommendations are missing to guide the use of antidepressants in bariatric surgery patients, in whom anatomical and physiological changes to the gastrointestinal tract, and weight loss may influence bioavailability of antidepressants in the immediate postoperative phase but also during long-term therapy. These changes in pharmacokinetics associated with depressive symptoms in the longer term postoperative course have not been analyzed in detail.

Our study aimed to investigate the pharmacokinetics of different SSRI/SNRI over a six-month course in people with morbid obesity who underwent bariatric surgery and define the effects on depressive symptoms (determined by the BDI questionnaire). Therefore, plasma levels of SSRI/SNRI were measured before bariatric surgery as well as 1 and 6 months after surgery, and were compared to plasma concentrations of SSRI/SNRI in patients on conservative therapy who served as a control group.

## Materials and methods

### Design

**Figure 1:**
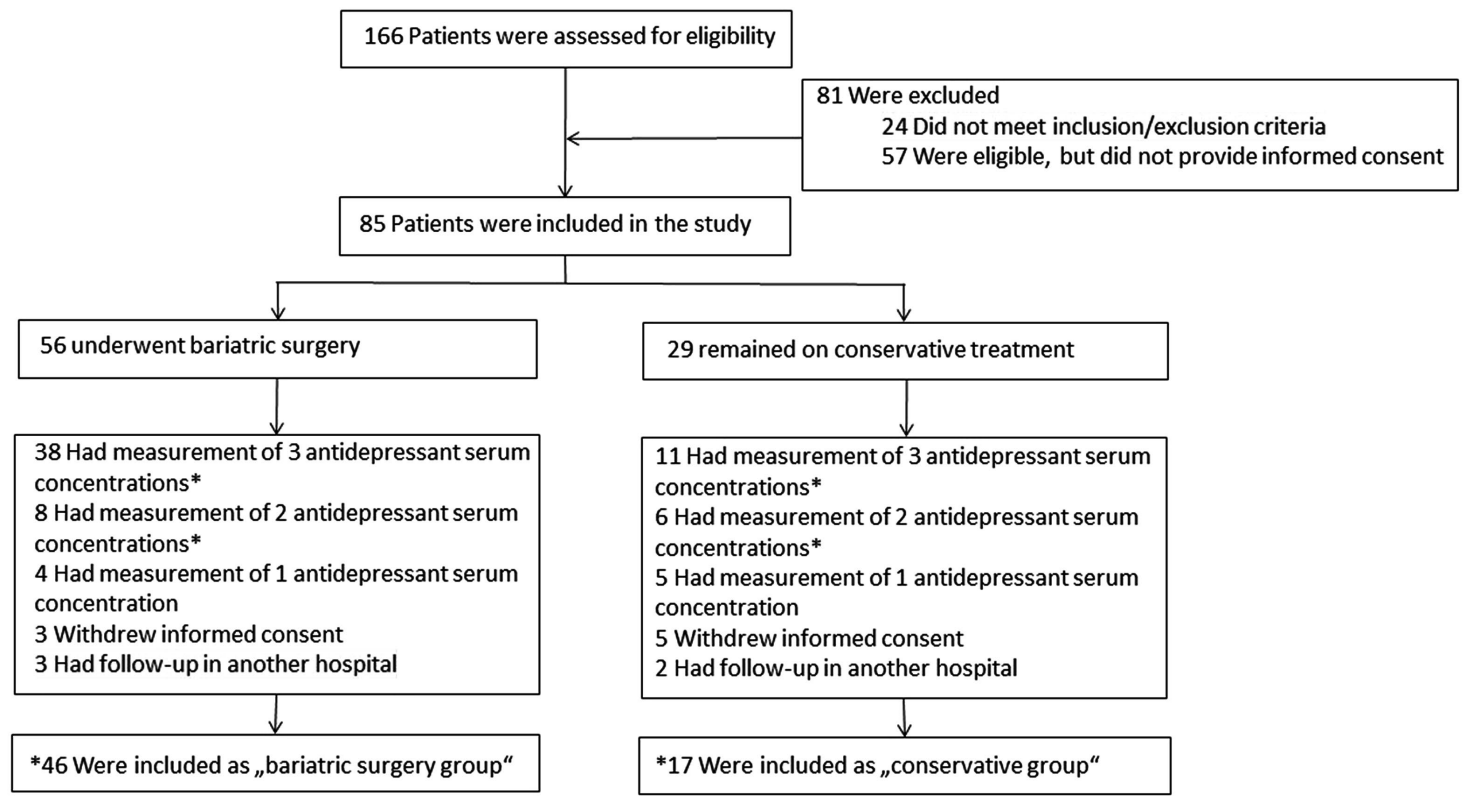
Enrollment and *inclusion of the final analysis.

Prospective multicenter study at three trial sites in Switzerland: the University Hospital Zurich (recruitment of 17 participants), the Inselspital Berne (32), and the Cantonal Hospital of Lucerne (14). Enrollment of patients began on February 1, 2017, and ended on January 31, 2020, and is shown in Figure 1.

Inclusion criteria were the following: age > 18 years, BMI >35, existing antidepressant drug therapy with SSRI or SNRI for 3 to 6 months (doses held stable during the observation period), no psychotherapy and written informed consent. Exclusion criteria were as follows: taking other psychotropic drugs than SSRI/SNRI, active drug addiction or psychosis, severe depression (BDI score > 30), suicidality (exclusion criteria for bariatric surgery) at the beginning of the study, gastrointestinal malabsorption, and pregnancy.

The study protocol was approved by the local institutional review board, and the Ethic Commission Nordwest-Central Switzerland (swissethics, EKNZ) ID 2017-00094, and the study was registered at ClinicalTrial.gov (NCT04928937). Written informed consent was obtained from each participant.

### Procedures

As part of the regular consultation at the obesity centers of the study sites obesity specialists decided about the appropriate therapy according to the patient’s will and to clinical criteria, and they assessed patients for participation in the study according to inclusion/exclusion criteria. Following a bariatric multidisciplinary team assessment, patients included in the bariatric surgery group underwent Roux-en-Y gastric bypass or sleeve gastrectomy representing two arms of the study. Patients in the conservative group (those who did not want to undergo surgery, or did not meet the formal criteria for bariatric surgery) were forming the third arm of the study. Subjects in the conservative therapy group had regular medical visits, nutritional counseling, physical therapy, anti-obesogenic medications and psychological coaching. Indication for surgery was given in accordance with the guidelines of the Swiss Society for the Study of Morbid Obesity and Metabolic Disorders (SMOB), recognized by the Federal Office of Public Health. Patients who met the inclusion criteria were asked to participate and were allocated according to the treatment option to the “bariatric surgery group” with the two subgroups “Roux-en-Y gastric bypass” and “sleeve gastrectomy,” or to the “conservative group.”

All participants filled the Beck Depression Inventory (BDI) questionnaire. Blood samples were taken three times: before surgery or at the beginning of the conservative treatment, respectively (T0); at 4 to 6 weeks (T1), and 6 months (T2) after surgery and after initiation of conservative treatment, respectively. Measurements of anthropometric and additional laboratory parameters were performed during the planned visits at the obesity centers.

The BDI is a psychological test that measures severity of depressive symptoms. It is a self-assessment measure including 21 questions on a four-level scale with values from 0 to 3 in terms of occurrence and intensity, providing a sum value between 0 and 63. Based on this value, it is possible to determine whether the person is not depressed, minimally depressed, mildly depressed, moderately to severely depressed or severely depressed. The internal consistency (Cronbach’s alpha) is between 0.74 (healthy controls) and 0.92 (younger patients with depression, up to age 30 years), depending on the population studied (13). The internal consistency for the 21 items of the BDI in our group of study patients was as follows: Cronbach’s alpha was 0.92 at T0 (*n* = 64), and 0.94 at T1 (*n* = 50), and 0.95 at T2 (*n* = 41), respectively.

The BDI inventory forms were submitted and checked by a staff member of the obesity center, and evaluations were carried out by the center’s psychiatrist.

Blood samples were drawn at the predefined time period (corresponding to the presumed Tmax) after ingesting the SSRI/SNRI. Measurements of SSRI/SNRI plasma levels were performed in an external laboratory (Analytica Medizinische Laboratorien, Zurich, Switzerland). Samples were kept at −80°C for up to 6 months and then sent to the laboratory. Analyses were performed in a steady state situation, i.e., after five elimination half-lives of the administered active substance. A capillary gas chromatographic-mass spectrometric method was applied to measure plasma following single oral doses. This method requires the extraction of 1 ml of plasma for the detection of 1 μg/L of drug. High performance liquid chromatography (HPLC) simultaneously quantified the drug and its major metabolites. Additional laboratory data were measured as part of the standardized follow-up after bariatric surgery: fasting insulin and glucose to determine HOMA-IR (Homeostasis Model Assessment of Insulin Resistance, i.e., fasting insulin-fasting glucose product divided by 22.5; normal ratio < 2.5).

BDI Scores and SSRI/SNRI plasma levels were entered into a GCP-compliant database (SecuTrial, iAS, Berlin, Germany).

In order not to compromise the validity of the study, the treating psychiatrists were instructed not to change the SSRI/SNRI dosage during the course of the study. In case of psychiatric emergencies, a psychiatric team was available to care for the patients.

### Statistical analysis

A sample size of 15 subjects per study arm was calculated sufficient to detect an effect size of 0.4 between the groups with a power of 80%, and a significance level of 5%. Statistical analyses were performed using GraphPad Prism version 8.0.1 for MacOS X, GraphPad Software, La Jolla, CA, USA.

Data is expressed as means and SD, unless stated otherwise. The paired T-test was used to assess normally distributed data (checked by the Kolmogorov–Smirnov test). The non-parametric Mann–Whitney test was applied otherwise. Group comparisons were performed with the Student’s T test and chi-squared test, respectively. Linear regression analysis were used to detect the relationship between the plasma concentrations of SSRI/SNRI, and outcome variables like weight, BMI, and BDI. All *p* values were two-tailed, and a *p* < 0.05 was considered statistically significant in the above tests.

## Results

A total of 63 participants were included in the study and completed the 6 months follow-up. There were 46 subjects in the bariatric surgery group (i.e., 20 participants in the gastric bypass group, and 26 in the sleeve gastrectomy group), and 17 in the conservative group. Participants’ baseline characteristics were similar with respect to anthropometric data, co-morbidities, BDI score, and antidepressant treatment (Table 1).

**Table 1:** Baseline characteristics of participants, undergoing bariatric surgery (*n* = 46), and on conservative treatment (*n* = 17).

	Bariatric surgery (*n* = 46)	Conservative (*n* = 17)	*p*
Age (years)	44.1 ± 12.5	40.5 ± 11.6	0.31
Sex (M/F)	11/35	3/14	0.59
BMI (kg/m^2^)	42.4 ± 5.7	43.1 ± 11.0	0.76
BDI	12.9 ± 8.0	14.7 ± 9.4	0.056
Treated comorbidities			
Diabetes mellitus type 2 (*n*)	4 (8%)	2 (11%)	0.71
Hypertension (*n*)	23 (50%)	4 (23%)	0.06
Dyslipidemia (*n*)	6 (13%)	2 (11%)	0.89
OSAS (*n*)	24 (52%)	6 (35%)	0.23
Smoking (*n*)	14 (30%)	4 (23%)	0.59
HOMA	5.7 ± 2.9	6.2 ± 2.3	0.67
Antidepressant treatment			
Citalopram	5^a^	1	
Escitalopram	12	5	
Fluoxetin	10	3	
Venlafaxin	8	3	
Duloxetin	6	2	
Paroxetin	1	0	
Sertralin	3	2	
Vortioxetin	1	0	
Bupropion	1^a^	1	

a One patient was treated with Citalopram and Bupropion.

Data are given as mean ± SD.

Patients’ characteristics were compared using Student’s *t*-test, and Chi-squared test for binary data.

OSAS, Obstructive Sleep Apnoea Syndrome.

HOMA, Homeostasis Model Assessment of Insulin Resistance (fasting insulin-fasting glucose product divided by 22.5. Normal < 2.5)

**Figure 2:**
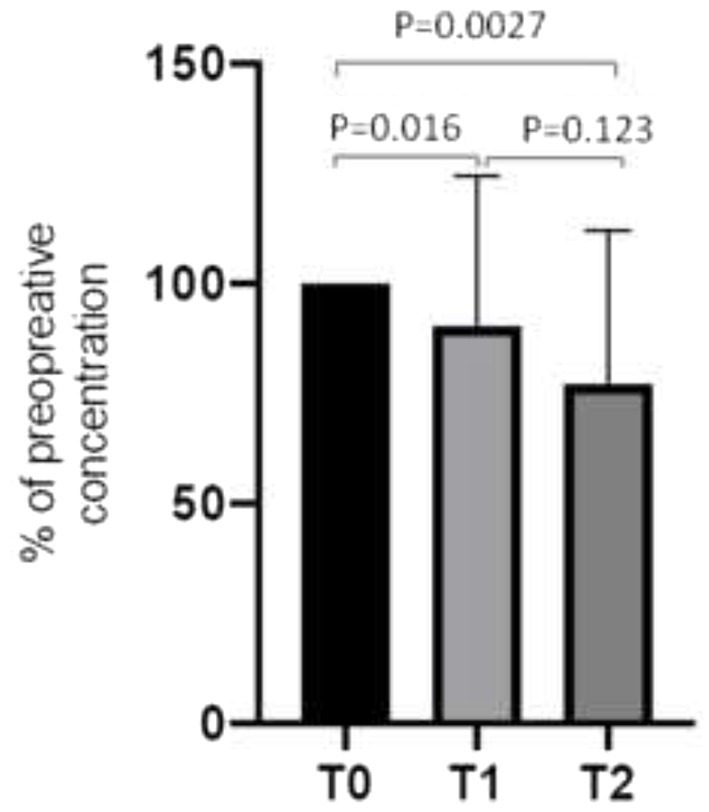
Plasma concentration of antidepressants SSRI/SNRI in patients undergoing bariatric surgery (*n* = 46): before (T0), 1 month (T1), and 6 months (T2) after bariatric surgery. To compare the plasma concentrations of the different SSRI/SNRI preoperative levels are set to 100%, and the values on T1 and T2 (μg/L, pmol/L, and mmol/L, respectively) are converted to %. Data are mean ± SD.

**Figure 3:**
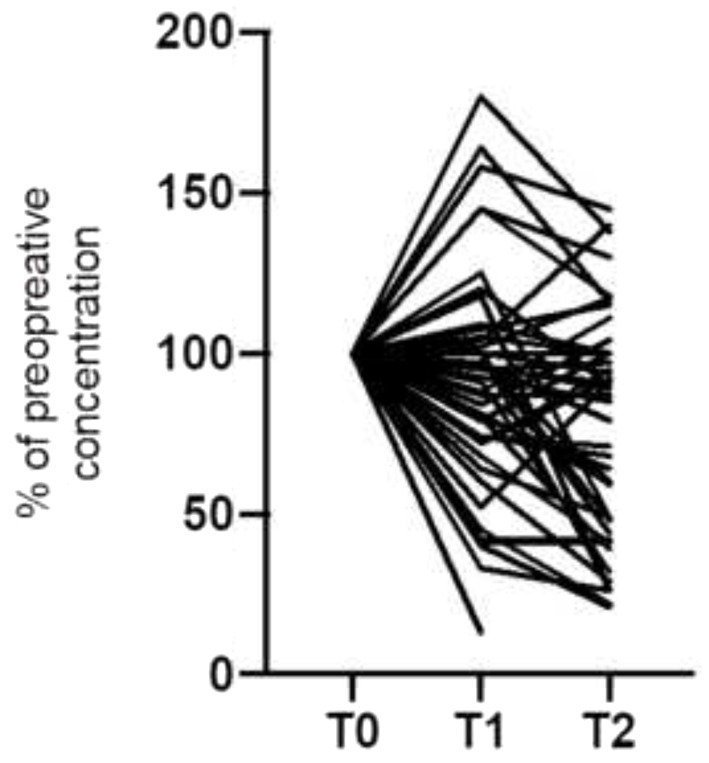
Individual course of the plasma concentration of antidepressants SSRI/SNRI in patients undergoing bariatric surgery (*n* = 46): before (T0), 1 month (T1), and 6 months (T2) after bariatric surgery.

**Figure 4:**
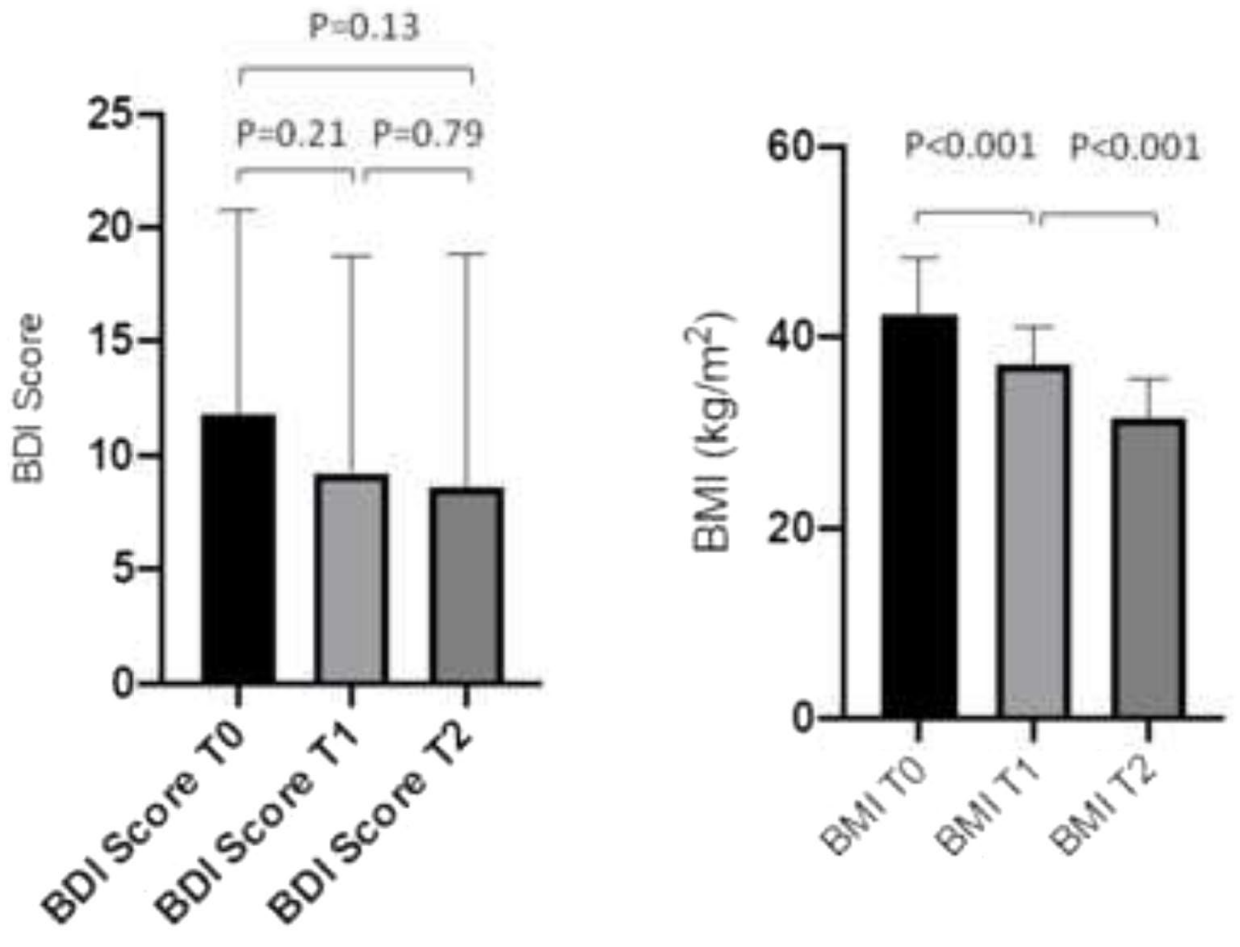
BDI score and BMI at beginning of the study (T0), and 1 month (T1), and 6 months (T2), after bariatric surgery (*n* = 46). Data are mean ± SD.

SRI/SNRI plasma concentrations dropped significantly in the bariatric surgery group from T0 (at the beginning of the study and before bariatric surgery) to T2 (6 months after bariatric surgery) by 24.7% (95% confidence interval [CI], −36.8 to −16.6, *p* = 0.0027): from T0 to T1 (4 weeks after bariatric surgery) SSRI/SNRI plasma concentrations decreased by 10.5% (95% CI, −22.7 to −2.3; *p* = 0.016), and from T1 to T2 by 12.8% (95% CI, −29.3 to 3.5; *p* = 0.123), respectively (Figure 2). There was a varied individual course of the plasma concentrations (Figure 3). Plasma concentrations of SSRI/SNRI remained unchanged throughout the 6 months follow-up in the conservative group (−14.7, 95% CI, −32.6 to 1.7; *p* = 0.076). However, the median [IQR] plasma concentrations of SSRI/SNRI in the conservative group were 100% [80; 132.5] at T1, and 90% [44; 115] at T2, indicating a trend toward statistical significance after 6 months of follow-up (i.e., T0 to T2: *p* = 0.076, 95% CI, −32.6 to 1.7), and a significance T0 to T2 = 0.036 (95% CI, −0.68 to −0.02, respectively). There was a significant loss of weight in the bariatric surgery group between T0 to T1 (BMI reduction by 5.3 kg/m^2^, 95% CI, −7.5 to −2.9; *p* < 0.001), and again to T2 (BMI reduction by 5.9 kg/m^2^, 95% CI, −7.6 to −3.7; *p* < 0.001). The mean %EBMIL in the bariatric surgery group after 6 months (T0 to T2) was 62.7% (95% CI, 54.2 to 69.1) and the mean %EWL was 55.6% (95% CI, 48.9 to 61.8). There was no significant change in the BDI score during follow-up (−2.9, 95% CI, −7.4 to 1.0; *p* = 0.13; Figure 4). In the conservative group, BMI (*p* = 0.20, 95% CI, −13.3 to 3.0), and BDI (*p* = 0.57, 95% CI, −14.1 to 8.0) remained unchanged during the six-month follow-up. Clinical outcomes with respect to SSRI/SNRI plasma concentrations, weight change, and change of BDI scores were similar in the subgroups undergoing gastric bypass surgery and sleeve gastrectomy, respectively (Table 2). In order to identify subgroups in which changes of the SSRI/SNRI levels would be associated with clinical outcomes linear regression analysis were performed.

**Table 2:** Comparison of clinical outcome in patients undergoing bariatric surgery: laparoscopic Roux-en-Y gastric bypass (RYGB) (*n* = 20) vs. sleeve gastrectomy (SG) (*n* = 26).

	RYGB	SG	*p*
SSRI/SNRI concentration T1 (%)	80.0 ± 43.7	93.1 ± 25.9	0.26
SSRI/SNRI concentration T2 (%)	73.8 ± 36.2	75.1 ± 35.1	0.91
BMI T1 (kg/m^2^)	36.3 ± 2.9	37.8 ± 4.5	0.26
BMI T2 (kg/m^2^)	30.5 ± 3.5	32.9 ± 4.6	0.26
Δkg	−27.0 + 19.5	−24.1 ± 14.2	0.55
BDI T1	12.2 ± 10.7	7.1 ± 4.1	0.07
BDI T2	11.2 ± 9.8	6.7 ± 7.7	0.13
Δ BDI (T0 – T2)	−1.7 ± 3.0	−1.3 ± 2.1	0.63

T1 denotes postoperative visit after 1 month, and T2 postoperative visit after 6 months, respectively.

Data are given as mean ± SD.

Student’s T test was used to compare outcome measures.

BMI, Body Mass Index [ratio between the body weight (kg) and height (m^2^)]

BDI, Beck Depression Inventory.

SSRI / SNRI, Selective Serotonin / Serotonin-Norepinephrine Reuptake Inhibitor.

However, plasma concentrations of SSRI/SNRI at T1 and T2, did not show any correlation to BDI 162 score (*r* = −0.13; 95% CI, −0.4 to 0.2, *p* = 0.42, and *r* = −0.15; 95% CI, −0.47 to 0.19, *p* = 0.38), and to BMI 163 (*r* = 0.15; 95% CI, −0.20 to 0.47, *p* = 0.39, and *r* = 0.23; 95% CI, −0.13 to 0.54, *p* = 0.21). Accordingly, changes of SSRI/SNRI concentrations did not correlate to BDI score < 15 vs. >15, and BMI <40 vs. >40, nor to age.

## Discussion

Our study investigating and comparing the pharmacokinetics of SSRI/SNRI in people with morbid obesity undergoing bariatric surgery showed that there was a significant postoperative decrease in plasma concentrations of SSRI/SNRI, however, without apparent effects on depressive symptoms.

SSRI/SNRI plasma concentrations dropped during the first 4 weeks after bariatric surgery; irrespective of surgery type (RYGB or SG) with further decrease thereafter during the 6 months of follow-up to about 75% of preoperative concentrations. The individual course of the postoperative SSRI/SNRI plasma level varied, and there was no correlation to the amount of weight loss, or change in BDI score.

Results of our study are consistent with those of previous studies of post-bariatric surgery patients describing the need to adjust antidepressant treatment, and the very different and unpredictable course of plasma concentrations of SSRI/SNRI (7–9, 14). In contrast to our study, all investigations and case series had a small number of patients without a control group, and the overall interpretation was limited to the RYGB. Based on these factors, the conclusions drawn from those studies were limited.

To the best of our knowledge, our prospective data is based on the longest duration and the largest number of patients so far, allowing to reliably represent the postoperative course of different SSRI/SNRI plasma concentrations. We hypothesized that plasma concentrations of SSRI/SNRI would correlate to the course of the depressive symptoms in obese patients undergoing bariatric surgery.

However, the severity of depressive symptoms in these patients remained unchanged, and there was no correlation to the significantly lower postoperative SSRI/SNRI levels. One possible explanation might be the inclusion of patients with only moderate depression (taking into account that severe depression and suicidality exclude bariatric surgery). However, considering the fact that in the conservative group both variables remained unchanged, there may be other variables compensating for the decreased resorption of SSRI/SNRI after bariatric surgery: in our study the preoperative weight and the postoperative weight loss were not associated with the course of depression, which fully concurs with a recent meta-analysis examining the effect of bariatric surgery on depression and anxiety (15). Although many studies report a postoperative improvement of depressive disorders (3, 16), others describe no change in anxiety disorders, possibly due to the elimination of eating as a way of coping with problems, or an increased risk of post-bariatric surgery depression compared to non-bariatric abdominal surgery controls (5, 17, 18). The anatomical and physiological alterations in the gastrointestinal tract after bariatric surgery may change bioavailability, pharmacokinetics, and drug dosing (19). There are a number of contributing factors like changes in gastric emptying time, an altered acid and bile acid environment, a decreased small intestine transit time, a reduced surface area, as well as modulation of the local microbiome (20, 21). By controlling tryptophan metabolism, this altered microbiome may influence the brain-gut axis with serotonin as a key neurotransmitter (22, 23).

Which one of these mechanisms precisely restrict oral bioavailability after RYGB and SG are not determinable in our study, and further studies will be needed to elucidate the role of the different clinical, inflammatory and neurohormonal factors affecting the course of postbariatric surgery depressive symptoms. However, because of varying plasma concentrations of SSRI/SNRI after bariatric surgery, postoperative therapeutic drug monitoring may be of value (24), and in case of malabsorption, daily dosage increase, switching to liquid oral dosage forms, and dividing the daily dosage into several daily drug intakes are strategies that can be implemented (25).

One limitation of our study is the fact that we compared single-point estimated peak SSRI/SNRI blood concentrations in patients after bariatric surgery and on conservative treatment. Postbariatric peak SSRI/SNRI levels may underestimate the clinical efficacy because of the unpredictable faster or slower absorption of the drugs (26). However, blood samples were taken in a predefined short time window to get comparable intraindividual and interindividual courses of the concentrations. Moreover, there are nine different drugs that can be given in a non-controlled release form included in this study. Four of the drugs can also be given in a controlled-release form. Therefore, not distinguishing between the controlled release and non-controlled release form of SSRI/SNRI is a further limitation of the study. In addition, our study did not examine the pharmacokinetics of SSRI/SNRI in the area under the curve (AUC).

## Conclusion

The study data, based on the longest duration and the largest number of patients so far, reliably represent the postoperative course of different SSRI/SNRI plasma concentrations. As an important consequence of the study findings measuring postoperative SSRI/SNRI plasma concentrations is neither reliable in predicting the course of depression nor adequate in the guidance of adjusting the dose of SSRI/SNRI. Further studies will be needed to elucidate the role of the different clinical, inflammatory and neurohormonal factors affecting postbariatric SSRI/SNRI resorption. In conclusion, in patients undergoing bariatric surgery plasma concentrations of SSRI/SNRI significantly decrease by about 25%, mainly during the first 4 weeks postoperatively, albeit with a wide variation, and without any correlation to the severity of depressive symptoms or weight loss.

## Data availability statement

The raw data supporting the conclusions of this article will be made available by the authors, without undue reservation.

## Ethics statement

The studies involving human participants were reviewed and approved by Ethikkommission Nordwest- und Zentralschweiz (EKNZ) Hebelstrasse 534,056 Basel. The patients/participants provided their written informed consent to participate in this study.

## Author contributions

PP and CH designed the study. PP, DK, AS, MS, AW, CM, GM, LH, SF, and CH recruited patients. PP, DK, SF, and CH analyzed the data. All authors contributed to the interpretation of data, drafting and revising critically the different sections of the manuscript, gave approval to the final version of the manuscript, and contributed to the work according to the Definition of Authorship as on the Submission Guidelines of the Frontiers Journal.

## Funding

This research received a grant from the clinical research funds of the Luzerner Kantonsspital LUKS, Department of Medicine (non-profit support).

## Conflict of interest

The authors declare that the research was conducted in the absence of any commercial or financial relationships that could be construed as a potential conflict of interest.

## Publisher’s note

All claims expressed in this article are solely those of the authors and do not necessarily represent those of their affiliated organizations, or those of the publisher, the editors and the reviewers. Any product that may be evaluated in this article, or claim that may be made by its manufacturer, is not guaranteed or endorsed by the publisher.

## Abbreviations

AUC: Area Under the Curve
SSRI: Selective Serotonin Reuptake Inhibitor
SNRI: Serotonin Noradrenalin Reuptake Inhibitor
RYGB: Laparoscopic Roux-en-Y gastric bypass
SG: Sleeve gastrectomy
BDI: Beck Depression Inventory
BMI: Body Mass Index (weight kg / height m^2^)
GCP: Good Clinical Practice
SMOB: Swiss Society for the Study of Morbid Obesity and Metabolic Disorder
FOPH: Federal Office of Public Health
%EWL: Percentage of Excess Weight Loss
%EBMIL: Percentage of Excess Body Mass Index Loss
